# Mining for New Sources of Resistance to Powdery Mildew in Genetic Resources of Winter Wheat

**DOI:** 10.3389/fpls.2022.836723

**Published:** 2022-03-01

**Authors:** Valentin Hinterberger, Dimitar Douchkov, Stefanie Lück, Sandip Kale, Martin Mascher, Nils Stein, Jochen C. Reif, Albert W. Schulthess

**Affiliations:** ^1^Leibniz Institute of Plant Genetics and Crop Plant Research (IPK), Seeland, Germany; ^2^German Centre for Integrative Biodiversity Research (iDiv) Halle-Jena-Leipzig, Leipzig, Germany; ^3^Center for Integrated Breeding Research (CiBreed), Georg-August-University, Göttingen, Germany

**Keywords:** genome-wide association mapping, powdery mildew, wheat, resistance, candidate genes, detached leaf assay, phenotyping, microphenomics

## Abstract

Genetic pathogen control is an economical and sustainable alternative to the use of chemicals. In order to breed resistant varieties, information about potentially unused genetic resistance mechanisms is of high value. We phenotyped 8,316 genotypes of the winter wheat collection of the *German Federal ex situ gene bank for Agricultural and Horticultural Crops, Germany*, for resistance to powdery mildew (PM), *Blumeria graminis f. sp. tritici*, one of the most important biotrophic pathogens in wheat. To achieve this, we used a semi-automatic phenotyping facility to perform high-throughput detached leaf assays. This data set, combined with genotyping-by-sequencing (GBS) marker data, was used to perform a genome-wide association study (GWAS). Alleles of significantly associated markers were compared with SNP profiles of 171 widely grown wheat varieties in Germany to identify currently unexploited resistance conferring genes. We also used the Chinese Spring reference genome annotation and various domain prediction algorithms to perform a domain enrichment analysis and produced a list of candidate genes for further investigation. We identified 51 significantly associated regions. In most of these, the susceptible allele was fixed in the tested commonly grown wheat varieties. Eleven of these were located on chromosomes for which no resistance conferring genes have been previously reported. In addition to enrichment of leucine-rich repeats (LRR), we saw enrichment of several domain types so far not reported as relevant to PM resistance, thus, indicating potentially novel candidate genes for the disease resistance research and prebreeding in wheat.

## 1. Introduction

Crop production has an important socioeconomic dimension, as social problems arise when people suffer from hunger or volatile food prices (Bellemare, 2015). For this reason, maximizing and stabilizing the production of our agroecosystems is fundamental to social stability worldwide in an era of rapid anthropogenic climate change, and continued growth in global demand for agricultural products. One of the most important yield-limiting factors in crop production is poor plant health. Current estimates suggests that about 20% of potential global yield of major crops is lost due to plant diseases (Savary et al., 2019). Much of this loss is caused by fungal pathogens (Savary et al., 2019). Farmers have several options to protect their crops from fungal epidemics: choosing appropriate crop rotations, soil cultivation, nitrogen fertilization, as well as direct measures such as fungicides and selecting resistant crop varieties. However, the number of effectively active components is shrinking due to legislative restrictions due to hazards for the environment and public health. In parallel, fungicide resistances developed by pathogens (Chin et al., 2001; Lucas et al., 2015) shrink their effect. Breeding resistant crops has therefore become increasingly important in recent decades. However, some widely deployed resistances had short life spans (McDonald and Linde, 2002; Brown, 2015). This is due to the great potential of the pathogen to circumvent resistance and the vulnerability of current agronomic practices to epidemics (Olesen et al., 2000). Therefore, establishing durable genetic protection in crops requires the use of multiple resistance mechanisms within the global agroecosystems (Fabre et al., 2012).

Plant Genetic Resources (PGR) are thought to provide a rich reservoir of potentially untapped resistance genes. These genes may never have been used in modern breeding or abandoned in the breeding process. PGR is a collective term for older varieties, landraces, and wild ancestors. To access this reservoir and use it for resistance breeding, carriers of unexploited resistance to different diseases need to be identified. One of the pathogens relevant to wheat (*Triticum aestivum* L.) is *Blumeria graminis* f. sp. *tritici*, the causal agent of powdery mildew (PM): PM epidemics occur in temperate and maritime regions and cause severe damage (Cowger et al., 2012). *Blumeria graminis* is an obligate biotrophic ascomycete, as such, it shows close host-pathogen interaction. This led to the high host specialization which can be observed (Inuma et al., 2007; Parks et al., 2008; Liu et al., 2015). Because of its ability to reproduce asexually and sexually and to spread aerially, this fungus is highly successful in genetic adaptation and rapid dispersal. To investigate this important pathosystem, phenotypic and genomic data were collected from around 8,000 winter wheat (*T. aestivum L*.) accessions, representing almost the entire IPK winter wheat collection, and 171 modern wheat varieties. Phenotypic data were generated under controlled conditions using a high-throughput detached-leaf phenotyping facility called Macrobot (full name BluVision Macro) (Lück et al., 2020a,b). The genotypic data were recently collected using genotyping-by-sequencing (GBS) (Schulthess et al., 2021). The main question was whether there are significant marker-trait associations within the PGR panel that identify beneficial alleles associated with resistance. If these loci are fixed for alleles associated with susceptibility within the Elite panel, the beneficial alleles from the PGR are good candidates for prebreeding of PM resistance. Therefore, a genome-wide association study (GWAS) was conducted in the PGR to identify the putative beneficial alleles. The identified loci were then compared to known PM resistance genes, and putative candidate genes were identified based on the reference genome.

## 2. Materials Methods

### 2.1. Plant Material

The analyzed population comprised 8,316 winter wheat (*T. aestivum L*.) PGR maintained at the *Federal ex situ gene bank for Agricultural and Horticultural Crop Species*, Germany, hosted at the *Leibniz Institute of Plant Genetics and Crop Plant Research* (IPK), as well as 171 European winter wheat varieties representing the currently cultivated varieties in Central Europe (in the following denoted as the Elite panel). For each PGR, seed samples were provided by the gene bank and multiplied in a first step using two-row plots (for details, see Schulthess et al., 2021). At this time, one and in rare cases two representative ears per plot were isolated during flowering for controlled self-fertilization and harvested separately from the rest of the plot at maturity. Seeds produced from each selfed single ear were then multiplied in a single-row plot during the following crop season. Seeds harvested from the single-row plots corresponded to the genetically defined propagation material of PGR used for DNA extraction and phenotyping. For the Elite panel, seeds for these purposes were collected from the seed market or directly provided by the seed industry.

### 2.2. High-Throughput Phenotyping of Plant-Pathogen Interactions

**Figure 1:**
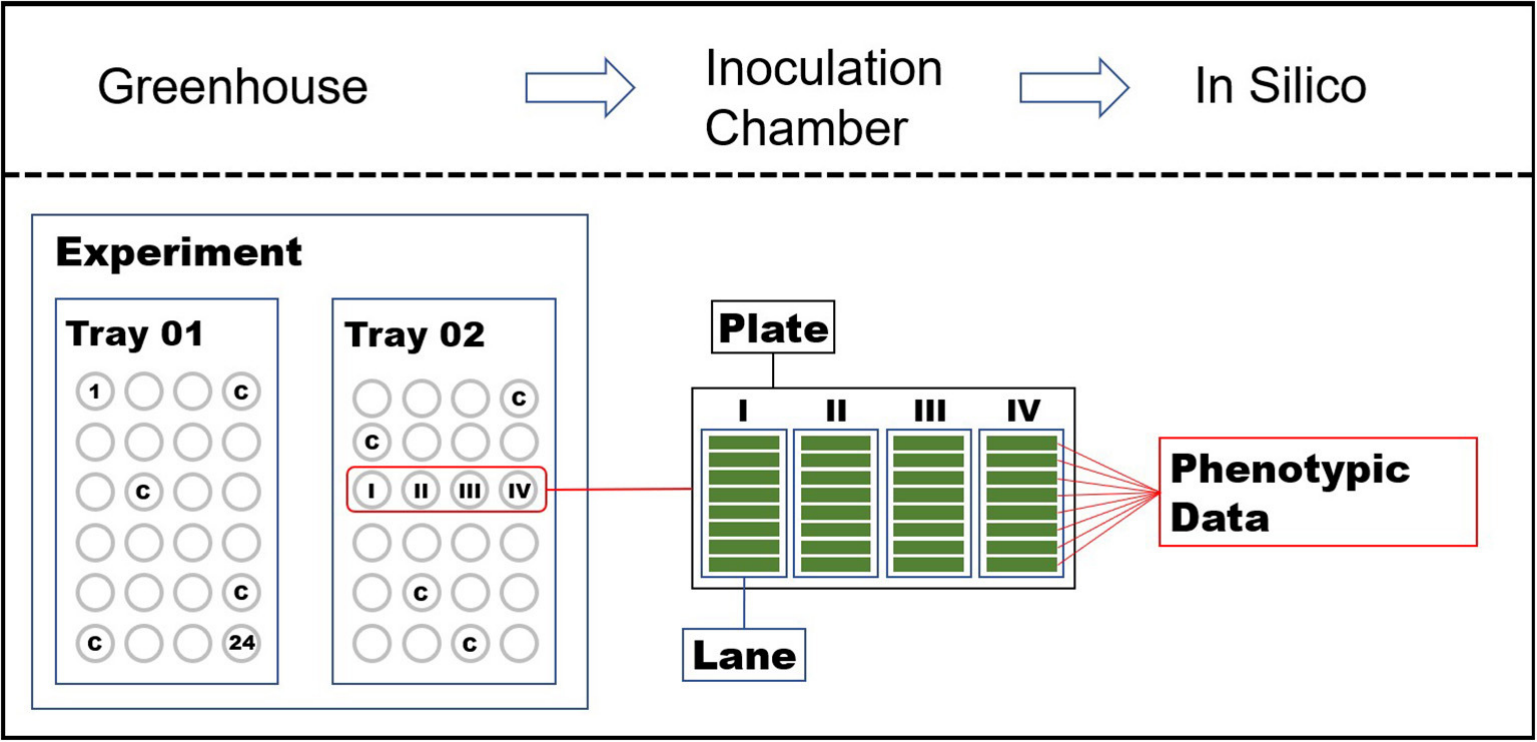
Schematic representation of the performed high-throughput detached leaf assay using the Macrobot Facility.

The phenotypic data was gathered using the Macrobot facility, a robotic platform for performing high-throughput semi-automatic detached leaf assays developed at the IPK Gatersleben (Lück et al., 2020a,b). Seedlings for the Macrobot assay were grown on trays comprising 6 × 4 slots in a greenhouse with standardized conditions at the IPK Gatersleben. For each genotype, 10 seeds were sown in one slot. Then, 15 days after sowing, the second leaves were harvested from seedlings. Up to eight leaves of different seedlings of the same genotype were collected. Leaf segments with a uniform length of 2 cm obtained from the middle part of the harvested leaves were mounted onto 4-well microtiter (MTP) agar-plates (1% water agar supplemented with 20 mg L^-1^ benzimidazole as a leaf senescence inhibitor), following the sowing pattern of the growth trays (see Figure 1 for a graphical illustration). The plates were inoculated in an inoculation tower with the highly virulent *Blumeria graminis* f. sp. *tritici* isolate FAL 92315 (see Supplementary Table 5). Spores from heavily PM infected plants were applied from the top of the inoculation tower using compressed air while the rotation of the platform with the open plates assured an even distribution of the spores. The inoculation density was controlled on microscope slides added between the plates and counted under a microscope after each portion of spores until the optimal spore density was reached (5–10 spores per mm^2^). The size of the rotatory platform allowed 12 MTPs with leaves harvested from two growth trays to be simultaneously inoculated, thus forming an experimental group. After inoculation, the plates were covered with the lids, transferred to an incubation chamber, and incubated for six days under controlled conditions (20°C, 60% RH constant, 16 h light μE m^-2^ s^-1^). Following incubation, multimodal images of the plates were automatically acquired and analyzed as described in Lück et al. (2020a) and Lück et al. (2020b). The disease severity was calculated as a percentage of the infected leaf area. In total, 8,487 genotypes were tested in 446 experiments. Each genotype was tested in two different experiments and up to eight individuals per genotype in each experiment. The susceptible cultivar KANZLER was used as running control by placing it in four out of the 24 slots of each growth tray.

### 2.3. Quality Assessment of Phenotypic Data

Detached leaf assays are artificial systems that are prone to certain inconsistencies due to the complexity of pathogen-host interactions and environmental influences, which can be reduced but never completely eliminated. Manual assessment of data quality was not feasible given the volume of the data, so an automatic, standardized quality pipeline was developed. To ensure high data quality, unreliable data at technical replicate, experimental, and biological replicate levels were identified and excluded from further analyses. The following data curation steps were performed: The first stage of outlier correction was based on the technical replications. In some cases, border-effects or leaves in a generally unhealthy state resulted in extreme values within the 3–8 technical replications. These were excluded by using 1.5 times the interquartile distance as the threshold. In a second step, the data were analyzed at the experiment level. Based on the susceptible control KANZLER, experiments with generally very low infection were excluded. Outliers were defined by using the 1.5 interquartile distance again as a threshold of the control genotype alone. If the mean or maximal value of the control of an experiment was below this threshold, the experiment was excluded from further investigation. The final quality check was based on the variance between the biological replicates, that is replicates of the same genotype in two different experiments. We implemented the outlier detection based on residuals as described in Anscombe and Tukey (1963) considering a nominal α level of 0.05. For this, we fitted the same model as used in Equation (1) and extracted the residuals. Datapoints exceeding the significant threshold were flagged as significant outliers and were excluded from further investigation. All computational methods were performed within R environment (R Core Team 2018 version 4.0.2. using R-Studio version 1.3.1056).

### 2.4. Estimation of the First- and Second-Degree Statistics

Variance components of the phenotypic data were estimated using a linear mixed model approach (Henderson, 1975).

The following mixed model was fitted to the percentage of infected leaf area data (y):


(1) $$\begin{array}{r} y=\mu +genotype+experiment+tray\left(experiment\right)+error \end{array}$$

where the common mean μ was treated as a fixed factor, whereas genotype, experiment, the tray nested within an experiment, and error effect were assumed as random factors. Best Linear Unbiased Estimations (BLUEs) of each genotype were computed using the same model as in Equation (1) but assuming the genotype factor as fixed. For variance components estimation and BLUEs computation, linear mixed models were solved using the ASReml-R package Version 4 (Bultler et al., 2009). The heritability was estimated as in the following equation:


(2) $$\begin{array}{r} h^{2}=\frac{\sigma_{G}^{2}}{\sigma_{G}^{2}+\frac{\sigma_{e}^{2}}{R}} \end{array}$$

where $\sigma_{G}^{2}$ is the genotypic variance, $\sigma_{e}^{2}$ is the residual variance while *R* represents the average number of replications per genotype. Heritability estimations from 500 different random samples containing 80% of the total number of genotypes were used to estimate the standard deviation of the heritability.

### 2.5. The Generation of GBS-Data

The study is based on genomic data presented in detail elsewhere (Schulthess et al., 2021). Briefly, genotyping was carried out at IPK facilities according to Poland et al. (2012) with some modifications. DNA was extracted from seedlings using established protocols by KWS LOCHOW GmbH. The DNA from each genotype was digested using *PstI* and *MspI* restriction enzymes (New England Biolabs) following Wendler et al. (2014) and ligated with adapters containing sample-specific barcode sequences. Later, processed barcoded DNA samples were pooled into groups of 540 genotypes in an equimolar amount to form a GBS library. Single-end (100 bp) sequencing was performed on Illumina HiSeq 2500. On average, 2.5 million reads were generated for each genotype, and these sequence reads were complemented with sequence information for 171 additional wheat varieties. Raw reads were trimmed in order to remove adapters and low-quality base calls. High-quality reads were then mapped against the reference genome sequence of Chinese Spring (IWGSC RefSeq v1.0), and SNP calling was carried out resulting in 1,646,929 SNP. The SNPs obtained were further filtered to keep those with homozygous genotype counts for each allele greater than 10, missing value rates smaller than 10%, and heterozygosity levels smaller than 1%. After filtering, information for 29,183 SNPs across 8,070 genotypes was available. To avoid introducing non-existent genetic variation into each pool, missing values were imputed separately for PGR and the Elite panel, respectively, using Random Forest implemented in the missForest R-package (Stekhoven and Bühlmann, 2012). Among genotypes with GBS profiles, 7,337 PGR-Isolates and 154 elite varieties had also curated Macrobot data available.

### 2.6. Genome-Wide Association Mapping

Genome-wide association mapping was performed using the R-Package rr-BLUP (Endelman, 2011), which uses a mixed model according to Yu et al. (2005):


(3) $$\begin{array}{r} y=X\beta +S\alpha +Zu+e, \end{array}$$

where y is a vector of the phenotypic data (BLUEs of infected leaf area); β is the fixed population mean; α is the fixed effect of the tested SNP; u is a random vector of polygene background effects and e is a vector of residual effects. X, S, and Z are incidence matrices relating y to β, α, and u. To account for population stratification and relatedness, a kinship matrix based on two times one minus Rogers' distance (RD) (Rogers, 1972) was estimated. Linear mixed models for GWAS were solved by using the “efficient mixed-model association” (EMMA)-algorithm (Kang et al., 2008) as implemented in the rrBLUP R-package (Endelman, 2011). Two significance thresholds were used for multiple-testing correction, both using a nominal level of test significance α = 0.05. Both thresholds were obtained using Bonferroni correction (α/n) but with two different n values. For the more restrictive threshold, n was defined as the total number of markers (29,183), while n of the less conservative threshold was calculated as the effective number of independent markers, M_eff_ (5,996) following Gao et al. (2008).

### 2.7. Secondary Analysis of the Significant Associations

For each significantly associated SNP, we compared the subpopulations defined by the two different alleles. The allele that occurred more frequently in the more resistant group was determined to be the beneficial allele. The significant SNP-trait associations were further analyzed using the Reference Genome Chinese Spring (IWGSC RefSeq v1.0). In a first step, we combined adjacent significant loci that were not interrupted by non-significant markers, aggregating marker-trait associations (MTA) to associated genomic regions.We also calculated the linkage disequilibrium (LD) for the SNPs within a defined region as the squared correlation coefficient (*r*^2^). We then collected the information of the predicted high-confidence genes and their predicted functional domains within these regions using the EnsemblePlants platform (Howe et al., 2019). The lines carrying the beneficial allele were defined as possible donor genotypes. We estimated the (*r*^2^) for the loci by fitting them individually into a simple linear model.

### 2.8. Domain Enrichment Analysis

To test whether certain domains were enriched around the significant associations, a domain enrichment analysis was performed. Using the Pearson's Chi-squared test, this analysis compares the relative abundance of the domains predicted in defined regions and their relative abundance in the whole genome. A significant region is defined by the distance between the next non-significant marker downstream and upstream of every significant marker-trait association. For this analysis, the reference genome annotation IWGSC v1.0 HC 20170706 was used. This version of the reference genome annotation contains 105,589 high confidence gene predictions. For all those genes, we predicted domains using PANTHER (Thomas et al., 2003), Gene3D (Lees et al., 2012), Pfam (Mistry et al., 2021), and PROSITE (Sigrist et al., 2012). First, for each domain type, the relative abundance was calculated. Then the relative abundance of each domain was calculated only using genes located in one of the significant regions.

### 2.9. Validation Panel Using Data From the German Federal Plant Variety Office

Phenotypic data were compared with data from field trials of the German Federal Plant Variety Office (Bundessortenamt, BSA). In these trials, the severity of the PM infection was rated by visual evaluation in nine categories from 1 (no infection) to 9 (severely infected). The data set contained phenotypic data from 2002 till 2019 for a total of 365 wheat varieties. Among these lines, 109 were also tested with the Macrobot. The Best Linear Unbiased Estimations (BLUE) were obtained by fitting the following linear mixed model:


(4) $$\begin{array}{r} y=\mu +Year+Genotype+Error \end{array}$$

where, y is the grade of infection, μ and *genotype* are the fixed general mean and fixed genotype effects, respectively, while year is the random effect of the year.

## 3. Results

### 3.1. Data Curation

**Figure 2:**
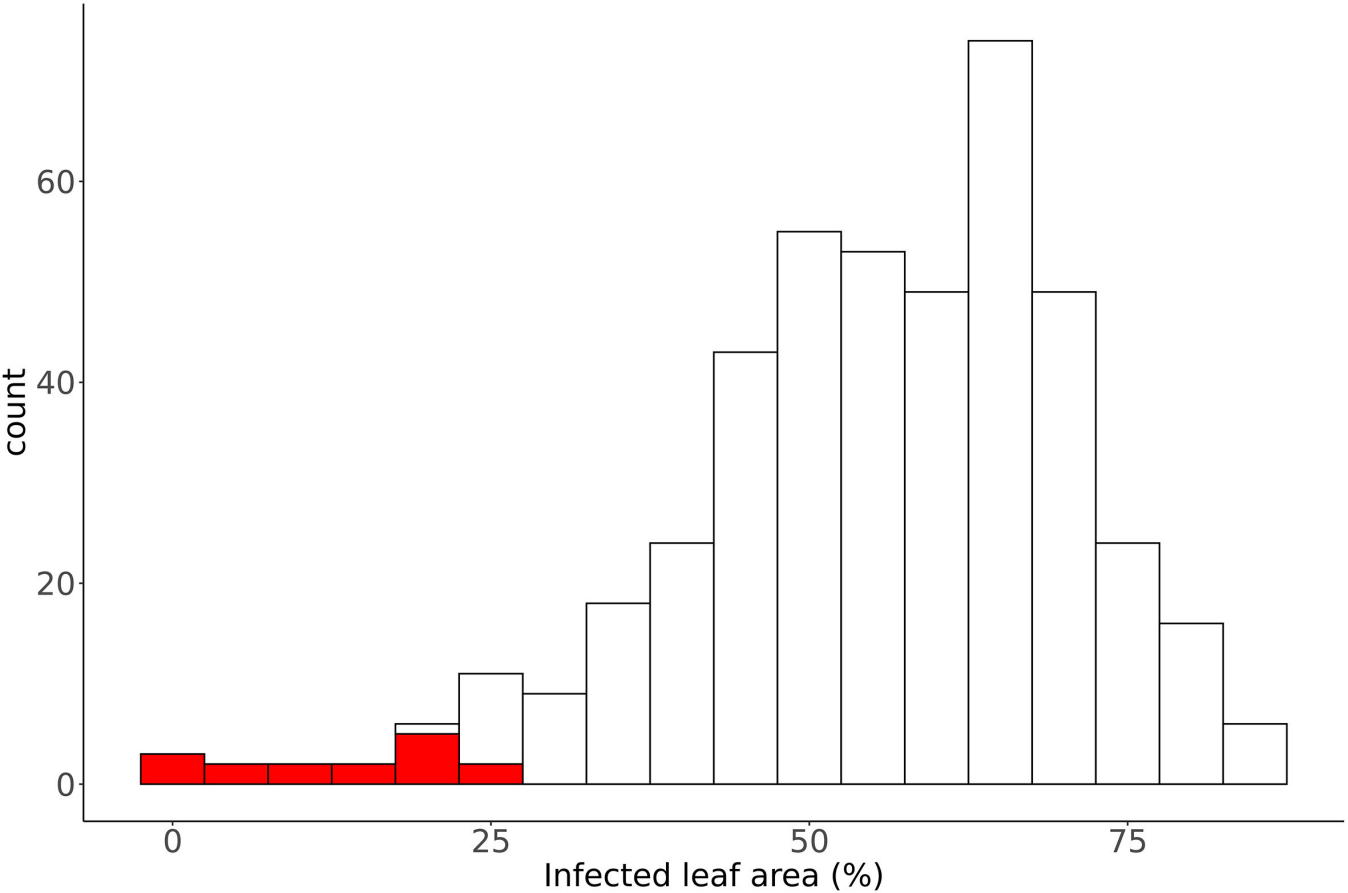
Distribution of the mean percentage of infected leaf area of the control genotype (Kanzler) of the 437 experiments. The red bars indicate detected outlier experiments.

Powdery mildew resistance screening of the winter wheat collection using the Macrobot system resulted in 158,469 data points. Each data point reflects one leaf measured. A total of 8,487 genotypes were phenotyped in 446 experiments. Data were analyzed in three hierarchical quality control steps. Two hundred ninety-nine of these data points were removed in the first step because they were considered outliers when comparing within technical replications. In the second step, 16 of the 446 experiments with a total of 5,571 data points were excluded because of the low infection rate of the susceptible control genotype (Kanzler) (Figure 2). In the third and final step, 697 data points were removed based on statistical outlier analysis among biological replications. Thus, 10,325 data points were excluded, representing only 6.5% of the total data. The overall data quality, as assessed by estimated heritability, was high. Data curation increased the heritability from 0.73 to 0.75 and reduced the variance component of the residuals (Table 1). We compared our data with results from the annual variety evaluation of the BSA. Macrobot data for PM resistance of 109 overlapping genotypes from the Elite panel showed a moderate correlation (*r* = 0.31). This correlation is within an expected range considering that our resistance assay is based on seedlings grown under greenhouse conditions and artificially inoculated with a specific PM isolate. In contrast, the BSA field trials are based on natural infections in field trials with locally adapted PM populations on adult plants.

**Table 1:** Variance components of the raw and the curated data set for powdery mildew infection measured as percentage of infected leaf area.

	**Raw data**		**Curated data**	
**Component**	**Estimation**	**SE**	**Estimation**	**SE**
Experiment	198.89	14.52	157.58	11.99
Experiment:Tray	25.46	2.29	26.03	2.35
Genotype	159.77	3.73	172.69	3.92
Residual	140.16	1.89	131.26	1.83
Heritability	0.73		0.75	
SD	0.005		0.005	

*The factor “Experiment” reflects which genotypes were infected and measured in one batch, “Tray” reflects all genotypes grown in a tray in the greenhouse. All components shown are significant at a threshold of 0.01. The standard deviation (SD) of the heritability was estimated from 500 heritability estimates using different random samples containing 80% of the total genotypes*.

### 3.2. European Elite Wheat Cultivars Show a Bimodal Distribution of Powdery Mildew Resistance

**Figure 3:**
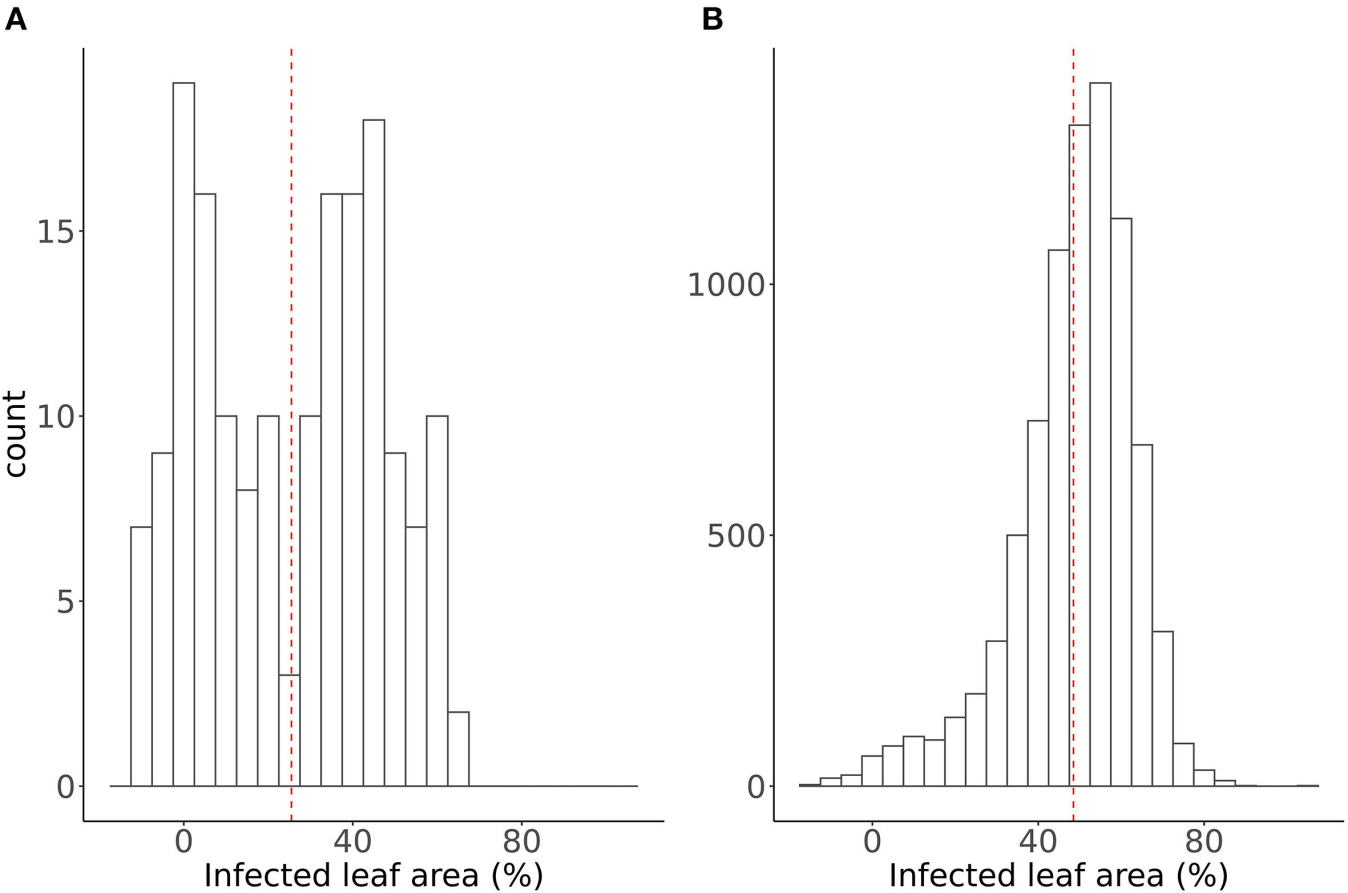
Distribution of Best Linear Unbiased Estimations (BLUEs) of the percentage of infected leaf area of **(A)** 170 European elite wheat cultivars and **(B)** 8,245 plant genetic resources (PGR). The dashed red line indicates the mean value.

The panel of 171 European elite wheat lines was on average more resistant than the 8,316 PGR. The mean percentage of infected leaf area of the elite lines and PGR were 25 and 48%, respectively. Nevertheless, 611 of the PGR were as resistant or even more resistant than the mean of the elite lines tested in our study (Figure 3). Furthermore, the Elite panel tested showed a binomial distribution of the BLUEs of infected leaf area. While our results illustrate the breeding progress in recent decades, the findings also highlight the need for the general implementation of PM resistance throughout the elite population and the potential of PGR as a source of this resistance.

### 3.3. Unused Molecular Genetic Diversity in Wheat Genetic Resources

**Figure 4:**
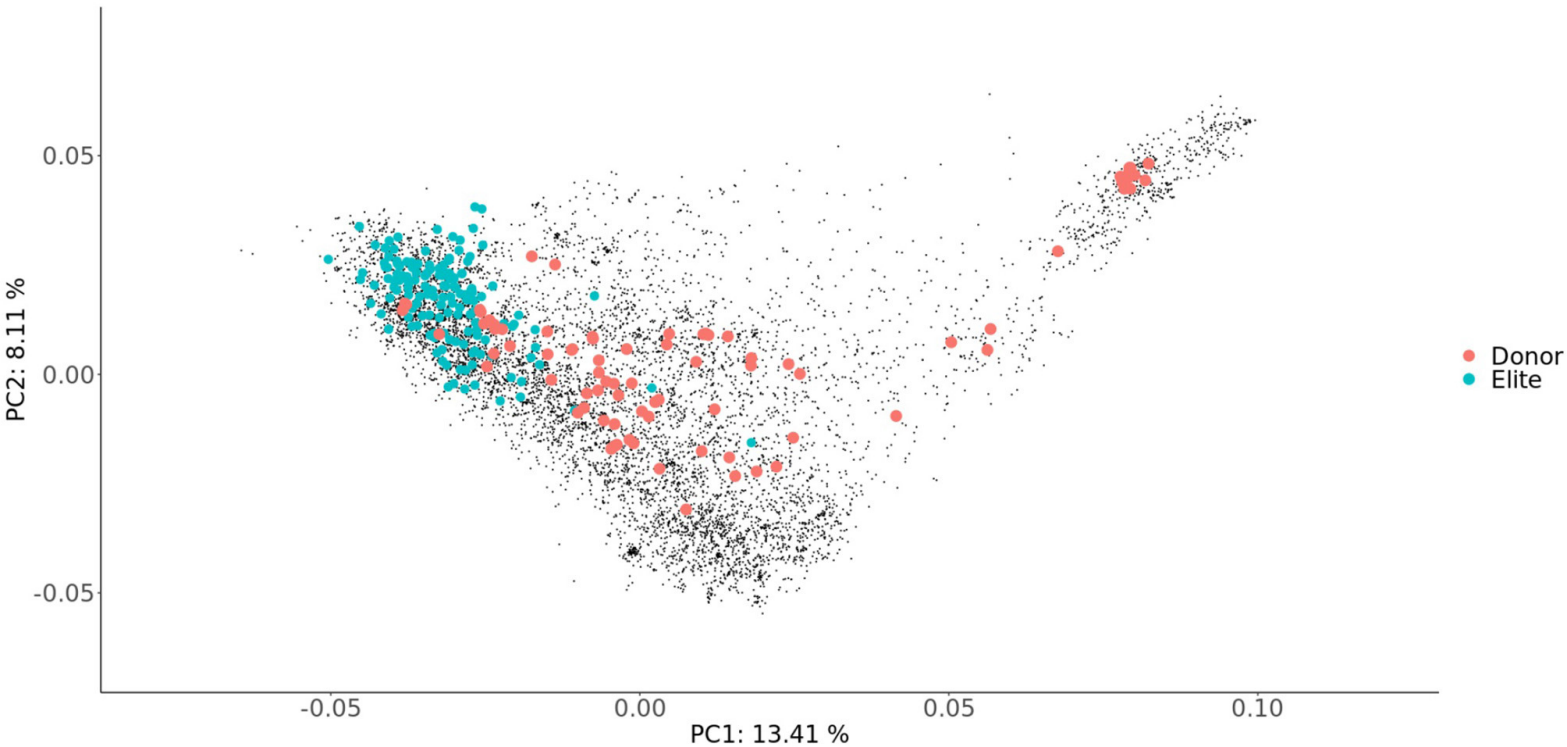
Principal coordinate analysis of the genotyped genotypes based on the Rogers' distances estimated using genotyping-by-sequencing profiles. PC1 and PC2 refer to the first and second coordinate, respectively. The proportion of explained molecular variance is indicated in parentheses. Blue dots denotes the elite cultivars, red ones the Donor genotypes carrying multiple beneficial alleles, gray ones the wheat genetic resources.

The principal coordinate analysis (PCoA) performed using the GBS data revealed a narrow relationship among the 170 elite cultivars compared to the genetic diversity of the PGR panel (Figure 4) and the erosion of genetic diversity in the elite line pool. The distribution of the lines carrying beneficial alleles that may serve as donor lines is broad, reflecting the total genetic variation. Most of them show moderate or high genetic distance to the Elite panel. Although genetic diversity does not automatically imply a favorable phenotypic diversity, it demonstrates the potential of untapped genetic variation stored within the gene bank.

### 3.4. Fifty Rare Loci for Powdery Mildew Resistance Identified

**Figure 5:**
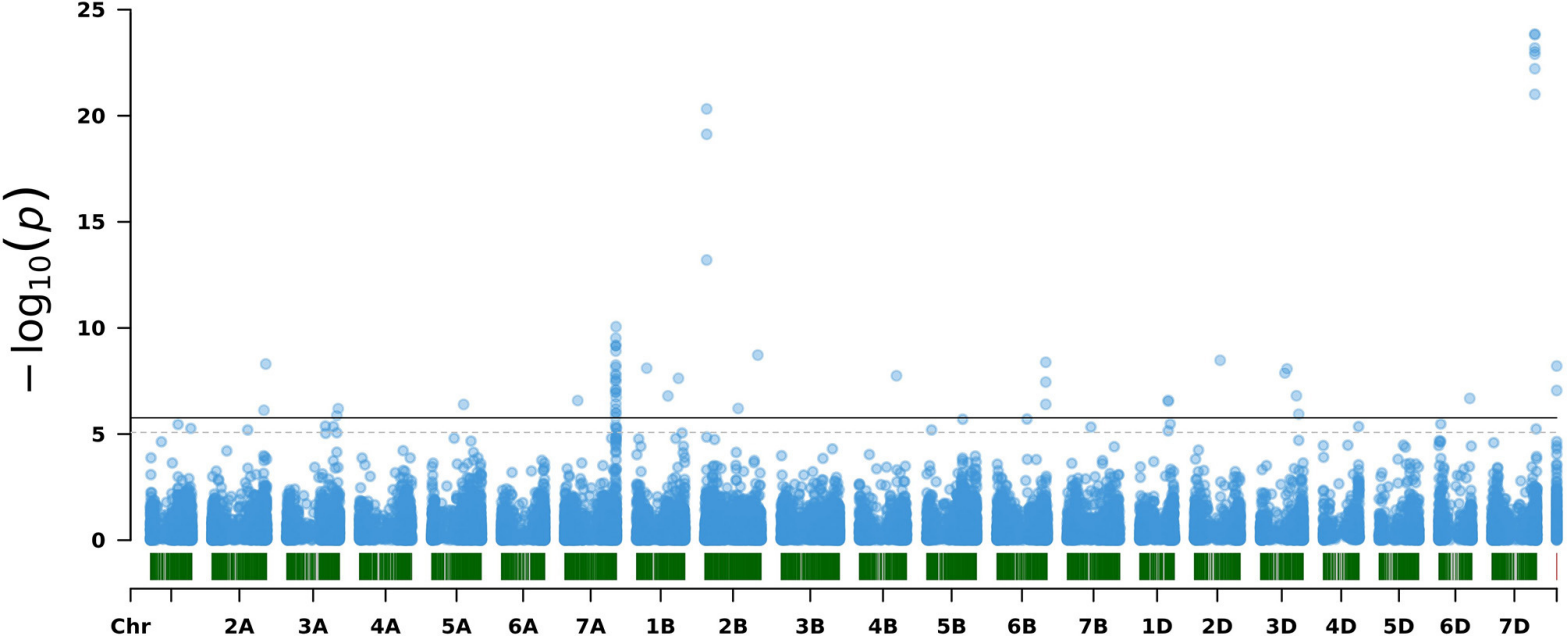
Manhattan plot of the genome-wide association scans for powdery mildew resistance using Best Linear Unbiased Estimations (BLUEs) of wheat Plant genetic resources (PGR). The solid and dotted lines indicate the significant thresholds corrected for multiple testing using the standard Bonferroni correction (Bonferroni, 1935) and the number of effective markers (Gao et al., 2008), respectively.

The GWAS performed based on the panel of wheat genetic resources resulted in 73 statistically significant marker-trait associations (MTA) located on 16 chromosomes (Figure 5; Tables 2–4; Supplementary Table 1). Two associated markers could not be assigned to a chromosomal location. The frequencies of the beneficial alleles (BAF) of the MTA were generally low in the population of PGR and absent or almost absent in the Elite panel. For the MTA, the mean frequency of the beneficial alleles was 0.004 in the Elite population. The ratios of explained variance (*R*^2^) of the 73 MTA ranged from 0.18 to 2% with mean R^2^ of 1.1%. The 73 MTA were combined into 51 resistance loci based on flanking non-significant markers. These markers were in high LD within the defined regions (mean *r*^2^ = 0.77). Individual loci spanned from 8 bp to almost 12 Mbp with a mean size of 0.8 Mbp, reflecting the wide variation in marker density of the subgenomes. Within all significant regions, 396 high confidence genes were predicted in the Wheat Reference Genome version 1.0 (Alaux et al., 2018). The number of genes per significant region varied from 0 to 50 (Supplementary Table 1), reflecting the differences in region sizes. For promising genes (Supplementary Table 1), we proposed KASP marker templates based on the reference genome for further use in research and breeding (Supplementary Table 6). To support the identification of candidate genes linked to markers significantly associated with mildew resistance, we performed domain enrichment analysis and observed highly significant enrichment of LRR domains in the identified significant regions as well as enrichment in other types of domains such as defensin, F-box-like domains among others (Supplementary Table 3). At this point, further detailed molecular genetic work is needed to reveal the genes and mechanisms behind the 51 discovered loci.

**Table 2:** Significant marker-trait associations (MTA) for powdery mildew resistance (PM-R) in winter wheat identified in a population of plant genetic resources (PGR) within the Wheat A-Genome.

**Chr**	**Published PM-R-Genes**	**MTA**	**BAF PGR**	**BAF Elite**	**-log_10_(*p*-value)**	***R* ^2^**
1A	Pm3a-r (3,9,94), Pm223899 (80), Pm17 (4,22), Pm25 (24)	1	0.9617	1	5.45	0.00002
		2	0.0031	0	5.27	0.013
2A	Pm4a-e (10,23,76,75,77) Pm50 (47), PmPS5A (31) PmLK906 (58) Pm65 (64), Ml92145E8-9 (68)	3	0.0033	0	5.19	0.006
		4	0.0031	0	6.12	0.013
		5	0.0015	0	8.3	0.007
						
3A	Pm44 (42)	6	0.0098	0	5.38	0.005
		7	0.0063	0	5.35	0.007
		8	0.0014	0	5.88	0.003
		9	0.003	0	6.2	0.014
5A	Pm55 (2), pm2026 (57)	10	0.0038	0	6.4	0.013
7A	Pm1a-e (3,4,7) Pm9 (4) Pm37 (35) Pm59 (1) PmU (54) Mlm2033 (56) Mlm80 (56) Pm60a-c (65,92)	11	0.0098	0	6.58	0.012
		12	0.0097	0.01	7.84	0.012
		13	0.0094	0.01	7.53	0.011
		14	0.0097	0.01	6.94	0.013
		15	0.0101	0.01	7.78	0.012
		16	0.0097	0.01	7.07	0.013
		17	0.0101	0.01	9.14	0.013
		18	0.0117	0.01	9.14	0.01
		19	0.0098	0.01	6.43	0.013
		20	0.0074	0.01	6.98	0.011
		21	0.0071	0.01	8.25	0.011
		22	0.0078	0.01	5.29	0.01
		23	0.0089	0.01	5.99	0.013
		24	0.0087	0.01	10.06	0.011
		25	0.0074	0.01	6.74	0.009
		26	0.0018	0.01	5.29	0.009

*BAF is the frequency of the advantageous alleles within the PGR population (n = 7,510) and the elite population (Elite, n = 170). Sources: (1) Tan et al., 2018, (2) Zhang et al., 2016, (3) Briggle and Sears, 1966, (4) Hsam et al., 1998, (7) Singrün et al., 2003, (9) Zeller et al., 1993, (10) McIntosh et al., 1979, (22) Hao et al., 2015, (23) Hao et al., 2008, (24) Shi et al., 1998, (31) Zhu et al., 2005, (35) Perugini et al., 2008, (42) Alam et al., 2011, (47) Mohler et al., 2013, (54) Qiu et al., 2005, (56) Yao et al., 2007, (57) Xu et al., 2008, (58) Niu et al., 2008, (64) Li et al., 2019, (65) Zou et al., 2018, (68) Yu et al., 2018, (75) McIntosh et al., 1979, (76) Li et al., 2017, (77) Ullah et al., 2018, (80) Li et al., 2018, (92) Zhao et al., 2020, (94) Jurkowski and Bujak, 2019*.

**Table 3:** Significant marker-trait associations (MTA) for powdery mildew resistance (PM-R) in winter wheat identified in a population of plant genetic resources (PGR) within the Wheat B-Genome.

**Chr**	**Published PM-R-Genes**	**MTA**	**BAF PGR**	**BAF PGR**	**–log_10_(*p*-value)**	***R* ^2^**
1B	Pm8 (4) Pm32 (6) Pm39 (36) Pm28 (27)	27	0.004	0	8.1	0.022
		28	0.0034	0	6.8	0.022
		29	0.0042	0	7.63	0.023
						
2B		30	0.0038	0.03	20.32	0.022
		31	0.0033	0	6.21	0.022
		32	0.0015	0	8.72	0.01
4B		33	0.0035	0	7.75	0.016
5B	Pm66 (83) Pm30 (29) Pm36 (34)	34	0.0029	0	5.7	0.015
		35	0.0027	0	5.19	0.015
						
6B	Pm11 (18), Pm12 (50), Pm14 (19), Pm15 (19), Pm20 (16), Pm54 (22), pmHYM (85), Pm27 (26)	36	0.0014	0	5.71	0.004
		37	0.0014	0.05	7.45	0.005
						
						
7B	Pm40 (14,38), Pm47 (45), Pm5a-e (5,11,12), pmDHT (70) Mlxbd (13,86), PmSGD (69), PmBYYT (82)	38	0.0037	0	5.33	0.006
						
						
						
						
						

*BAF is the frequency of the advantageous alleles within the PGR population (n = 7,510) and the elite population (Elite, n = 170). Sources: (4) Hsam et al., 1998, (5) Hsam et al., 2001, (6) Hsam et al., 2003, (11) Law and Wolfe, 1966, (12) Huang et al., 2003, (13) Huang et al., 2000, (14) Marone et al., 2013, (16) Friebe et al., 1994, (18) Tosa et al., 1988, (19) Tosa and Sakai, 1990, (22) Hao et al., 2015, (26) Järve et al., 2000, (27) Peusha et al., 2000, (29) Liu et al., 2002, (34) Blanco et al., 2008, (36) Lillemo et al., 2008, (38) Luo et al., 2009, (45) Xiao et al., 2013, (50) Jia et al., 1996, (69) Xu et al., 2018, (70) Qie et al., 2019, (83) Li H. et al., 2020, (82) XU et al., 2018, (83) Li H. et al., 2020, (85) Fu et al., 2017, (86) Jin et al., 2020*.

**Table 4:** Significant marker-trait associations (MTA) for powdery mildew resistance (PM-R) in winter wheat identified in a population of plant genetic resources (PGR) within the Wheat D-Genome.

**Chr**	**Published PM-R-Genes**	**MTA**	**BAF PGR**	**BAF Elite**	**-log_10_(*p*-value)**	***R* ^2^**
1D		39	0.0059	0	5.15	0.011
		40	0.0015	0	6.59	0.014
		41	0.0014	0	5.48	0.013
2D		42	0.0015	0	8.48	0.01
3D		43	0.0018	0	7.88	0.012
		45	0.002	0	6.8	0.014
		46	0.0023	0	5.93	0.011
4D		47	0.0046	0	5.35	0.009
6D		48	0.0029	0	5.48	0.01
		49	0.0027	0	6.68	0.007
7D	Pm38 (37), Pm29 (6,28) Pm19 (6)	50	0.0026	0	22.22	0.009
		51	0.0098	0.03	5.24	0.005

*BAF is the frequency of the advantageous alleles within the PGR population (n = 7,510) and the elite population (Elite, n = 170). Sources: (6) Hsam et al., 2003, (28) Zeller et al., 2002, (37) Spielmeyer et al., 2005*.

### 3.5. Identification of 11 Novel Resistance Loci

Identifying novel resistance loci in the PGR not currently used in European wheat breeding is one central research question. We compared the 51 MTA identified in this study with the extensive literature on reported PM resistance genes. We identified 11 MTA mapping to chromosome arms for which, to our knowledge, no PM resistance gene has been reported yet (Supplementary Table 4). These 11 MTA map to Chr3AL (MTA6-9), Chr7AS (MTA11), Chr3DL (MTA43-46) Chr4DL (MTA47), and Chr6DL (MTA49). The individual MTA differ significantly in size of the associated physical regions and the number of predicted genes located therein. Detailed information on these MTA and the putative candidate genes has been compiled in Supplementary Table 1. Several susceptible alleles were fixed in the commercial lines, leaving the breeding pool without an internal source of those favorable alleles. The PGR genotypes can provide the necessary resistance donors to compensate for the missing beneficial alleles in the elite line pool (Supplementary Table 2).

## 4. Discussion

Apart from potential false positives associations, several factors limit the usability of GWAS and QTL analysis for downstream breeding and scientific applications. To transfer QTL mapped with biparental populations is difficult when the genetic backgrounds of mapping and breeding populations differ significantly (Melchinger et al., 1998; Utz et al., 2000; Malosetti et al., 2020). Another limitation to the transferability of results is population size and composition. The population must be of sufficient size to detect rare alleles or alleles with minor effects (Melchinger et al., 1998; Utz et al., 2000; Korte and Farlow, 2013). Currently used mapping populations for PM are mostly in sizes between 100 and 1,000 genotypes (Ullah et al., 2018; Leonova, 2019; Kang et al., 2020; Simeone et al., 2020). To overcome many of these problems and assess a broader diversity, for this study we pheno- and genotyped almost the entire winter wheat collection of the German Federal *ex-situ* Gene Bank and a set of 171 elite varieties currently used in Europe. Many GWAS studies are using a minor allele frequency threshold of 0.05. Although this value is accepted as a standard, it is an arbitrary value. In our case, working with a population of more than 8,000 genotypes, this threshold would result in a minimum of at least 400 genotypes carrying minor alleles, which is more than the typical size of populations used in association studies. Therefore, we decided to use a minor allele count of 10 to keep the rare alleles in the analysis while ensuring a solid data basis. This threshold should also ensure that rare alleles of the potentially highest relevance to breeders will not be lost in the analysis. The predicted phenotypic effects (*R*^2^) of the found MTA were very low compared to other published GWAS. However, the broad diversity, low MAF, and high number of MTA in this study compared to biparental populations, lead likely to a significant underestimation of *R*^2^. The next step in MTA evaluation will be to develop segregating biparental elite × PGR populations for the most interesting MTA. This will allow to validate MTA and their estimated effects and generate bridging germplasm for use in prebreeding.

### 4.1. Repeatable High Throughput Powdery Mildew Resistance Phenotyping

Host-pathogen interactions are a complex and dynamic process influenced by several biotic and abiotic factors. Naturally occurring infections can differ in population composition (Andrivon and De Vallavieille-Pope, 1993; Parks et al., 2009), and environmental factors (e.g., weather) play a critical role in their development (Cowger et al., 2012). Therefore, the use of experiment replications and the application of artificial infections can increase the precision in field phenotyping for resistance. Although this approach is feasible for smaller collections, its application to the entire PGR collection would require considerable efforts. Instead, the detached leaves assay combined with an automatic image analysis pipeline (Lück et al., 2020a) provided a high level of control over the environmental and biotic factors and ensured repeatable phenotypic data for the entire population at a reasonable cost in our study. The high observed heritability (0.75) support this expectation. Macrobot- and field data for PM resistance of 109 overlapping genotypes from the Elite panel showed a correlation of *r* = 0.31. Kang et al. (2020) observed a correlation of the PM resistance for 329 wheat varieties in the field and the greenhouse of 0.4–0.5. Tucker et al. (2006) reported an overall higher PM infection in the greenhouse than in the field using 293 recombinant inbred lines. In addition, when testing a BC1 double haploid population of 94 individuals, Mohler et al. (2013) observed correlations of 0.52 and 0.82 between adult plants in the greenhouse and in the field depending on the year. The latter study was based on a mixed pathogen population in the greenhouse and natural infection in the field. Moreover, high differences were also observed when comparing seedling and adult plant responses to PM (Wang et al., 2005; Jakobson et al., 2006; Mohler and Stadlmeier, 2019). Thus, we conclude that the method used in this study is less suitable for direct estimation of the genotype performance in the field, but rather provides the basis for the discovery of novel resistance mechanisms and crossing candidates for further trials. The Macrobot phenotyping provides a good proxy for fungal biomass (Lück et al., 2020b) and allows quantitative estimations of the fitness of a pathogen isolate on the host genotypes with only a small amount of seeds needed. Combined with a speed-breeding approach (Watson et al., 2018), this could significantly accelerate the breeding progress for resistance against PM. Another main difference to field conditions is the used pathotype. We used a single isolate, while the natural PM population structure is highly spatiotemporally diverse (Parks et al., 2009; Mascher et al., 2012) and responding to the deployment of resistance genes in the form of selection of favorable mutations and population shift (Andrivon and De Vallavieille-Pope, 1993). In this respect, the Macrobot system allows many genotypes to be tested against defined pathotypes, opening the opportunity to distinguish between monogenic R-gene resistance and durable race-nonspecific resistance mechanisms, thus assisting the integration of resistance alleles into elite varieties.

### 4.2. A Glimpse Into the Potential of the Presented MTA

Powdery mildew resistance is a complex and long-studied trait with more than 90 genes at more than 60 loci reported so far (McIntosh et al., 2017; Tang et al., 2018; Ullah et al., 2018; Supplementary Table 4). However, only 12 of them have been cloned to date: *Pm2* (Sánchez-Mart́ın et al., 2016), *Pm3* (Yahiaoui et al., 2004), *Pm4* (Sánchez-Mart́ın et al., 2021), *Pm5* (Xie et al., 2020), *Pm8* (Hurni et al., 2013), *Pm17* (Singh et al., 2018), *Pm21* (Cao et al., 2011; He et al., 2018; Xing et al., 2018), *Pm24* (Lu et al., 2020), *Pm38* (Krattinger et al., 2009), *Pm41* (Li M. et al., 2020), *Pm46* (Sánchez-Mart́ın et al., 2016), *Pm60* (Zou et al., 2018),—reviewed also by Simeone et al. (2020). To demonstrate the potential value of the 51 resistance loci discovered in this study, we show a detailed examination of four of them (MTA5, MTA30, MTA37, and MTA50) which are interesting for different reasons. Briefly, MTA5 and MTA30 are co-localized with the well-known Pm4 and Pm26 locus, respectively. MTA37 is special because it is in contrast to the other MTA enriched in the tested Elite panel. MTA50 exhibit the highest estimated −log_10_(*p*-value). The localization of the MTA was assumed to correspond to the Chinese Spring reference genome to enable comparison of the detected associations with known published genes. Of course, it is possible that the physical position of the MTA in the individual genotypes within the population differs from the reference genome, e.g., due to translocations, inversions, InDels, transposable elements, and other structural rearrangements of the genome (Appels et al., 2018; Bariah et al., 2020). Resistance genes in particular are subject to abundant structural variation, which decreases the mapping precision of associations and makes gene cloning thus more difficult (Dolatabadian et al., 2020; Nsabiyera et al., 2020). Most of the significant associations found were mapped to chromosome arms for which genes or QTLs associated with PM resistance have been previously reported. However, this does not necessarily mean that the associated marker is related to these genes. In many cases, the available sources do not include physical localization at a higher resolution than the chromosome arm or chromosome (Tables 2–4; Supplementary Table 1).

#### 4.2.1. MTA5—A Novel Receptor-Like Kinase at the Pm4 Locus

TraesCS2A02G563900, one of the five predicted genes in MTA5, encodes for a Receptor-like protein kinase, carrying multiple LRR-, a protein kinase domain, a transmembrane domain, and a signal peptide. The role of Receptor-like kinases resistance proteins has been well described in rice (*Xa21* and *LysM*; Joris et al., 1992; Song et al., 1995), wheat (*TaRLK1* and *-2, Pm21*; Chen et al., 2016; He et al., 2018), and Arabidopsis (*EFR*; Kaku et al., 2006). Based on the domain structure of TraesCS2A02G563900 we propose it as a candidate for PM resistance underlying MTA5. To elucidate whether TraesCS2A02G563900 is an already described resistance gene, we investigated the MTA5 locus in more detail. McIntosh et al. (1979) and Ma et al. (2004), and later Hao et al. (2008) and others linked the *Pm4* locus to the long arm of Chr2A. The *Pm4* locus contains multiple alleles of the *Pm4* gene (a-e) (Ullah et al., 2018). Schmolke et al. (2011) genetically mapped the *Pm4* locus between flanking markers *Xgwm356* (753472205) and *Xbarc122* (766164161), which, according to the reference sequence, are also flanking MTA5 here (Supplementary Figure 1). This could suggest that this MTA5 candidate is a member of the *Pm4* locus. *Pm4* has been cloned recently by Sánchez-Mart́ın et al. (2021), showing also that the here used reference sequence (Chinese Spring) does not contain a close homologue to *Pm4*. It is therefore unlikely that TraesCS2A02G563900 is Pm4. Furthermore, due to the low MAF of MTA5 in the tested populations, it is unlikely that MTA5 represents the *Pm4b* allele that has already been used excessively in resistance breeding (Bundessortenamt, 2018). In addition, considering the virulence structure of the used isolate (Supplementary Table 5), we think it is also unlikely to detect *Pm4a* or *b* among the screened material. The used PM isolate FAL92315 is heavily virulent for *Pm4a* and *Pm4b*, so we assume no phenotypic resolution using this isolate. *Pm4e* maps within the *Pm4*-QTL region flanked by *Xgwm356* and *Xbarc122* (Ullah et al., 2018) but is located 1,699,142 bp away from the MTA5 locus. Another candidate (TRITD2Av1G295560) was recently detected in the same region by Simeone et al. (2020) as a QTL for PM adult plant resistance in durum wheat. In conclusion, we presume that TraesCS2A02G563900 is a new putative candidate for resistance against PM, unused in current European elite varieties and not *Pm4*.

#### 4.2.2. MTA37—A Resistance Locus Enriched in the Elite Panel

The significant MTA's favorable alleles were mainly rare in the PGR and almost or entirely absent in the Elite panel. An exception to this pattern is MTA37. While rare in the PGR, the three significant SNPs underlying MTA37 were enriched in the Elite panel (see Table 3). The three significant SNPs underlying MTA37 are located on Chr6BL. Four high-confidence genes were predicted in this region: two (TraesCS6B02G429800 and TraesCS6B02G429900) are promising candidates because of their F-Box and LRR domains. F-Box/LRR-domain proteins have been described as required for elicitor-triggered HR response (van den Burg et al., 2008) and associated with stripe rust resistance in wheat (Yin et al., 2018; Zhang et al., 2018). The MTA37 region co-localizes in Chinese Spring with *Pm54* (Hao et al., 2015) and *PmG3M* (Xie et al., 2012). These genes have not been cloned yet, so there is no information about their structure. However, it has been reported that *PmG3M* is derived from an *T. dicoccoides* introgression (Xie et al., 2012). It was also recently investigated functionally and shown to provide a post-haustorial defense mechanism (Wei et al., 2020). The other reported genes for Chr6B tend to map to the short arm of Chr6B (*Pm11, Pm12, Pm20*, and *Pm07j126*) or in the case of *Pm27* 143,716,930 bp downstream of MTA37. The elite lines carrying this locus (“Arktis,” “Chiron,” “Gustav,” “Sailor,” “Halvar,” and others) are highly resistant in our experiments and field trials of the BSA. This is an indication that this locus may also confer PM resistance under field conditions. We, therefore, suspect that MTA37 and its candidates are more likely *PmG3M, Pm54*, or an unknown gene that has been selected by breeding.

#### 4.2.3. MTA 30—Putative Candidate for Pm26

Compared to the two examples above, MTA30 is a large locus spanning 1,360,110 bp on Chr2BS (Chr2B:26573946-27934056). This is due to the low marker density distal to the 3 significant markers which determine the locus. Twenty-seven genes were predicted for the region. Some of them show a classical NB-LRR structure (TraesCS2B02G054900, TraesCS2B02G055000, TraesCS2B02G055300) others encode SWEET genes (TraesCS2B02G055700-TraesCS2B02G056100). Sugar transporters are known susceptibility genes for biotrophic pathogens (Chen et al., 2010; Gupta, 2020). Nevertheless, it is not possible to further narrow down the possible candidates without further evaluations. The genetic marker *Xcau516*, shown to co-segregate with *Pm26* (Liu et al., 2012), maps precisely to MTA30. Therefore, we conclude that among all resistance genes described on Chr2B so far (*Pm06, Pm26, Pm33, Pm42, Pm49, Pm51, Pm52, Pm57*, and *Pm68*), MTA30 is most likely *Pm26*, i.e., a resistance gene introgressed from *T. timopheevii*. Further evaluation is needed to elucidate whether the elite lines carrying the positive allele of MTA30 (Anapolis, Atomic, KWSBarny, and Panorama) carry this introgression. All four lines show meager infection rates in both our assay and BSA field trials. This suggests that this locus could also confer PM resistance under field conditions.

#### 4.2.4. MTA 50—Major Effect MTA on Chr7DL Encodes for a Homeobox-Like Transcription Factor

The highest -log_10_(*p*-value) in our study was estimated for the MTA50 on Chr7DL. This MTA comprises seven highly significant SNPs. Within this region, two genes were predicted. The first one is predicted to encode a Homeobox-like protein, and the other shows no known domains. Homeobox-like domain proteins are transcription factors and were already described to play a role in resistance against PM in wheat (Liu et al., 2008). Transcription factors can be an interesting starting point for exploring new resistance mechanisms by investigating the expression patterns of contrasting genotypes when infected. This could lead to a better understanding of the regulation of immunity reaction and the identification of new resistance candidates. *Pm19* and *Pm29* were mapped to Chr7DS before. The only information available for *Pm19* is its location on Chr7D with no specification of the chromosome arm (Lutz et al., 1995); we, therefore, cannot exclude the possibility that MTA50 refers to *Pm19*. *Pm29* was mapped with AFLP markers, which we could not map to the reference genome, so the only information available is that this gene lays distal to the markers *PSR129* and *XksuD2* at Chr7DL (Zeller et al., 2002). MTA50 also maps to the distal part of this chromosome arm, so we could not exclude that MTA50 refers to *Pm29*. Therefore, we conclude that MTA50 most likely refers to a transcription factor that could be synonymous with *Pm19* or *Pm29*. Further evaluations using PM pathotypes selective to *Pm19* and *Pm29* in a population segregating for MTA50 could answer whether *Pm19, Pm29*, and MTA50 are independent PM resistance genes or not. The four examples shown above demonstrate the potential of the here published loci as a resource for cloning R-genes and prebreeding approaches. The same holds from our perspective true for the identified novel resistance loci on Chr3AL, Chr3DL, Chr4DL, and Chr6DL. We hope to encourage further investigations using the here presented material and data.

### 4.3. Resistance Donors for Powdery Mildew Resistance Prebreeding

The many rare associations which are negatively fixed in the tested Elite panel show the huge potential in the tested PGR for modern wheat resistance breeding. We identified several accessions which carry multiple MTA from our experiment (see Supplementary Table 2). For all those MTA, the resistance conferring alleles are not present within the Elite panel and contribute significantly to the resistance against powdery mildew in our assay using the PGR. Those donors can be ordered from the Federal *ex situ* gene bank for Agricultural and Horticultural Crop Species in Gatersleben for prebreeding and scientific usage. To investigate the functionality in the field and use the described MTA in prebreeding we recommend the use of the Cadenza and Kronos TILLING Resources.

### 4.4. Agronomical Implications

These and other studies have attempted to elucidate the genetic basis of resistance and isolate resistance genes for subsequent use in agriculture. However, with few exceptions, the identification and introgression of a single major QTL have not been confirmed to be a sustainable solution to ensure resistance as discussed previously in terms of the “boom-and-bust cycle” of resistance genes (McDonald and Linde, 2002). Furthermore, stacking many minor QTL is very laborious and time-consuming and even these quantitative resistances erode over time. Therefore, smarter strategies and interdisciplinary efforts are needed to protect our agroecosystems in the coming decades. One approach is to access novel resistance genes and mechanisms and optimize their management. McDonald (2014) proposed using dynamic diversity contrary to static diversity, based on deployment of single major-gene- or quantitative resistance, or constant R-gene pyramiding. However, this promising concept requires a significant increase in genotype diversity and breeding for populations composed of many genotypes. Diversifying the selection pressure exerted by the resistance genes used is one of the key strategies. As proposed by Hafeez et al. (2021), integrated approaches for creating a comprehensive wheat R-gene atlas should help cope with the challenge of the continuously changing pathogen populations. Another possibility would be establishing a classification in resistance genetics equivalent to the FRAC (Fungicide Resistance Action Committee) classification of fungicides. This classification groups all fungicides according to their mechanisms of action, allowing the farmer to diversify selection pressures. Growers could use such a classification to rotate and mix cultivars for resistance, as with fungicides. Regardless of the selected approach, the first and crucial step in the direction of resistance diversification is to reveal and exploit the genetic diversity stored in the gene banks worldwide. This information will allow growers and breeders to employ the new resistance genes even without knowing the mechanisms behind them.

## 5. Conclusion

In summary, 51 MTA regions containing promising candidate genes, involving NB-LRR type R-genes but also other types of resistance-related genes were identified by GWAS. Eleven of these MTA were mapped to chromosome positions for which no PM resistance genes have previously been reported. This indicates the high potential of the tested gene bank material as a source for novel PM resistance. KASP markers have been proposed for all MTA, allowing further functional validation of the identified MTA. We also provide a list of donors for the MTA for use in prebreeding, as well as a list of candidate genes for PM resistance.

## Data Availability Statement

The raw data supporting the conclusions of this article will be made available by the authors, without undue reservation.

## Author Contributions

AS, JR, MM, and NS designed the study. DD generated phenotypic data. SL performed the image analysis. SK and NS generated the GBS-Data. VH curated the data, performed the analyses, and wrote the manuscript with the input of all other authors. All authors contributed to the article and approved the submitted version.

## Funding

The experimental work was supported by the German Federal Ministry of Education and Research within the GeneBank2.0 Project (Grant Nos. FKZ031B0184B and FKZ031B0184A).

## Conflict of Interest

The authors declare that the research was conducted in the absence of any commercial or financial relationships that could be construed as a potential conflict of interest.

## Publisher's Note

All claims expressed in this article are solely those of the authors and do not necessarily represent those of their affiliated organizations, or those of the publisher, the editors and the reviewers. Any product that may be evaluated in this article, or claim that may be made by its manufacturer, is not guaranteed or endorsed by the publisher.
