# Supplementary material for: Mining for New Sources of Resistance to Powdery Mildew in Genetic Resources of Winter Wheat
Source: Front Plant Sci. 2022 Mar 1;13:836723. doi: 10.3389/fpls.2022.836723 (PMC8922026; doi:10.3389/fpls.2022.836723)
Supplement: Supplementary file 2 [file Table_2.DOCX]

**Table S3:** Results of the domain enrichment analysis using the predicted domains of annotated genes within the MTA regions compared with their frequency in the overall genome.

| **Domain description** | **count in total** | **count in MTA regions** | **pValue** |
| --- | --- | --- | --- |
| ACT domain | 121 | 2 | 0.002451 |
| ATPase, F1/V1/A1 complex, alpha/beta subunit, N-terminal domain | 143 | 2 | 0.006554 |
| ATPase, F1/V1/A1 complex, alpha/beta subunit, nucleotide-binding domain | 165 | 2 | 0.013632 |
| ATPase, alpha/beta subunit, nucleotide-binding domain, active site | 121 | 2 | 0.002451 |
| Alfin | 55 | 2 | 1.66E-06 |
| Carbohydrate-binding, CenC-like | 99 | 2 | 0.000607 |
| Chitin-binding, type 1 | 198 | 2 | 0.030545 |
| Chitin-binding, type 1, conserved site | 176 | 2 | 0.018416 |
| Chromo-like domain superfamily | 77 | 2 | 7.09E-05 |
| Chromo/chromo shadow domain | 33 | 2 | 3.85E-10 |
| Clp ATPase, C-terminal | 143 | 2 | 0.006554 |
| Cyclin | 143 | 2 | 0.006554 |
| Cyclin, C-terminal domain | 132 | 2 | 0.004167 |
| Cyclin, N-terminal | 154 | 2 | 0.00969 |
| Cytokinin dehydrogenase 1, FAD/cytokinin binding domain | 187 | 2 | 0.024054 |
| Cytokinin dehydrogenase, C-terminal domain superfamily | 187 | 2 | 0.024054 |
| Defensin, plant | 99 | 2 | 0.000607 |
| Endochitinase-like superfamily | 198 | 2 | 0.030545 |
| F-box-like domain superfamily | 7524 | 19 | 0.036851 |
| FAD-linked oxidase-like, C-terminal | 209 | 2 | 0.037873 |
| Glutamate carboxypeptidase 2-like | 121 | 2 | 0.002451 |
| Glycoside hydrolase family 10 domain | 110 | 2 | 0.001303 |
| Glycoside hydrolase, family 19 | 187 | 3 | 4.04E-05 |
| Glycoside hydrolase, family 19, catalytic | 187 | 3 | 4.04E-05 |
| Glycoside hydrolase, family 32 | 231 | 5 | 5.15E-12 |
| Glycosyl hydrolase family 32, C-terminal | 253 | 5 | 5.86E-11 |
| Glycosyl hydrolase family 32, N-terminal | 231 | 5 | 5.15E-12 |
| Glycosyl hydrolase, five-bladed beta-propellor domain superfamily | 297 | 5 | 2.63E-09 |
| Heat shock protein 70 family | 165 | 3 | 9.33E-06 |
| Heat shock protein 70, conserved site | 154 | 3 | 3.85E-06 |
| Heat shock protein 70kD, peptide-binding domain superfamily | 165 | 3 | 9.33E-06 |
| Histidine phosphatase superfamily | 110 | 3 | 2.07E-08 |
| Histidine phosphatase superfamily, clade-1 | 77 | 3 | 9.98E-12 |
| IQ motif, EF-hand binding site | 539 | 4 | 0.003317 |
| Immunoglobulin E-set | 176 | 3 | 2.03E-05 |
| Jacalin-like lectin domain | 297 | 4 | 7.17E-06 |
| Jacalin-like lectin domain superfamily | 308 | 4 | 1.16E-05 |
| Knottin, scorpion toxin-like | 121 | 2 | 0.002451 |
| Leucine-rich repeat | 4312 | 24 | 3.63E-11 |
| Lysozyme-like domain superfamily | 187 | 3 | 4.04E-05 |
| MRG | 11 | 2 | 7.80E-26 |
| Neprosin | 209 | 3 | 0.000129 |
| Neprosin activation peptide | 132 | 3 | 4.30E-07 |
| PA domain | 429 | 4 | 0.000474 |
| Pentatricopeptide repeat | 22 | 4 | 1.30E-67 |
| Peptidase M28 | 143 | 2 | 0.006554 |
| Protein of unknown function DUF241, plant | 110 | 3 | 2.07E-08 |
| RNA binding activity-knot of a chromodomain | 22 | 2 | 2.05E-14 |
| SWEET sugar transporter | 132 | 5 | 6.17E-21 |
| Senescence regulator S40 | 154 | 2 | 0.00969 |
| Tapetum determinant 1 | 110 | 2 | 0.001303 |
| Transferrin receptor-like, dimerisation domain | 110 | 2 | 0.001303 |
| Zinc finger C2H2-type | 1188 | 8 | 2.56E-05 |
| Zinc finger, PHD-type, conserved site | 396 | 3 | 0.015327 |
