# Supplementary material for: Mining for New Sources of Resistance to Powdery Mildew in Genetic Resources of Winter Wheat
Source: Front Plant Sci. 2022 Mar 1;13:836723. doi: 10.3389/fpls.2022.836723 (PMC8922026; doi:10.3389/fpls.2022.836723)
Supplement: Supplementary file 3 [file Table_3.DOCX]

**Table S4a:** Powdery mildew resistance genes, their chromosomal location, source of identification and all in this study identified Marker Trait Associations (MTA) of winter wheat A-Genome.

| **CHR** | **Known genes** | | **MTA** | |
| --- | --- | --- | --- | --- |
|  | Short arm | Long arm | Short arm | Long arm |
| Chr1A | Pm3a-r (3,9,94)  Pm223899 (80) | Pm17 (4,22) |  | MTA1  MTA2 |
|  | Pm25 (24) | |  |  |
| Chr2A |  | Pm4a-e (10,23,76,75,77)  Pm50 (47)  PmPS5A (31)  PmLK906 (58)  Pm65 (64)  Ml92145E8-9 (68) |  | MTA3  MTA4  MTA5 |
| Chr3A | Pm44 (42) |  |  | **MTA6**  **MTA7**  **MTA8**  **MTA9** |
| Chr4A | Pm16 (20,21) | |  |  |
|  |  | Pm61 (81)  PmHHXM (90) |  |  |
| Chr5A | Pm55 (2) | pm2026 (57) |  | MTA10 |
| Chr6A |  | Pm21 (30,61,67)  Pm56 (60) |  |  |
| Chr7A |  | Pm1a-e (3,4,7)  Pm9 (4)  Pm37 (35)  Pm59 (1)  PmU (54)  Mlm2033 (56)  Mlm80 (56)  Pm60a-c (65,92) | **MTA11** | MTA12  MTA13  MTA14  MTA15  MTA16  MTA17  MTA18  MTA19  MTA20  MTA21  MTA22  MTA23  MTA24  MTA25  MTA26 |

**Table S4b:** Powdery mildew resistance genes, their chromosomal location, source of identification and all in this study identified Marker Trait Associations (MTA) of winter wheat B-Genome.

| **CHR** | **Known genes** | | | **MTA** | |
| --- | --- | --- | --- | --- | --- |
|  | Short arm | | Long arm | Short arm | Long arm |
| Chr1B |  | Pm8 (4)  Pm32 (6)  Pm39 (36) | | MTA27 | MTA28  MTA29 |
|  | Pm28 (27) | | |  |  |
| Chr2B | Pm26 (25)  Pm42 (40)  Pm49 (46) | Pm6 (14,15,66) Pm33 (31)  Pm51 (48) Pm52 (72)  Pm57 (49,87) Pm63 (62)  Pm64 (59) Pm68 (63)  PmQ (91) | | MTA30 | MTA31  MTA32 |
| Chr3B |  | Pm13 (51) Pm41 (39,64)  PmY39 (52) MlZec1 (55) | |  |  |
| Chr4B | Pm7 (6,16) | | |  | MTA33 |
|  |  | Pm66(83) | |  |  |
| Chr5B | Pm30 (29) | Pm36 (34) | | MTA34 | MTA35 |
| Chr6B | Pm11 (18)  Pm12 (50)  Pm14 (19)  Pm15 (19)  Pm20 (16) | Pm54 (22)  pmHYM (85) | |  | MTA36  MTA37 |
|  | Pm27 (26) | | |  |  |
| Chr7B | Pm40 (14,38)  Pm47 (45) | Pm5a-e (5,11,12)  pmDHT (70)  Mlxbd (13,86)  PmSGD (69)  PmBYYT (82) | |  | MTA38 |

**Table S4c:** Powdery mildew resistance genes, their chromosomal location, source of identification and all in this study identified Marker Trait Associations (MTA) of winter wheat D-Genome.

| **CHR** | **Known genes** | | **MTA** | |
| --- | --- | --- | --- | --- |
|  | Short arm | Long arm | Short arm | Long arm |
| Chr1D | Pm24 (13,74) | Pm67 (78) |  | MTA39  MTA40  MTA41 |
|  | Pm10 (17) | |  |  |
| Chr2D |  | Pm43 (14,41) |  | MTA42 |
| Chr3D |  |  |  | **MTA43**  **MTA44**  **MTA45**  **MTA46** |
| Chr4D |  |  |  | **MTA47** |
| Chr5D | Pm2a-c (3,8,84)  PmSub (88)  PmJM23 (79)  Pm10V-2 (71)  PmCH1357 (73)  Pm46 (44)  Pm48 (93) | Pm34 (32)  Pm35 (33)  PmY201 (53)  PmY212 (53) |  |  |
| Chr6D | Pm45 (43) |  | MTA48 | **MTA49** |
| Chr7D | Pm38 (37) | Pm29 (6,28) |  | MTA50  MTA51 |
|  | Pm19 (6) | |  |  |
