# Supplementary material for: Mining for New Sources of Resistance to Powdery Mildew in Genetic Resources of Winter Wheat
Source: Front Plant Sci. 2022 Mar 1;13:836723. doi: 10.3389/fpls.2022.836723 (PMC8922026; doi:10.3389/fpls.2022.836723)
Supplement: Supplementary file 5 [file Table_5.DOCX]

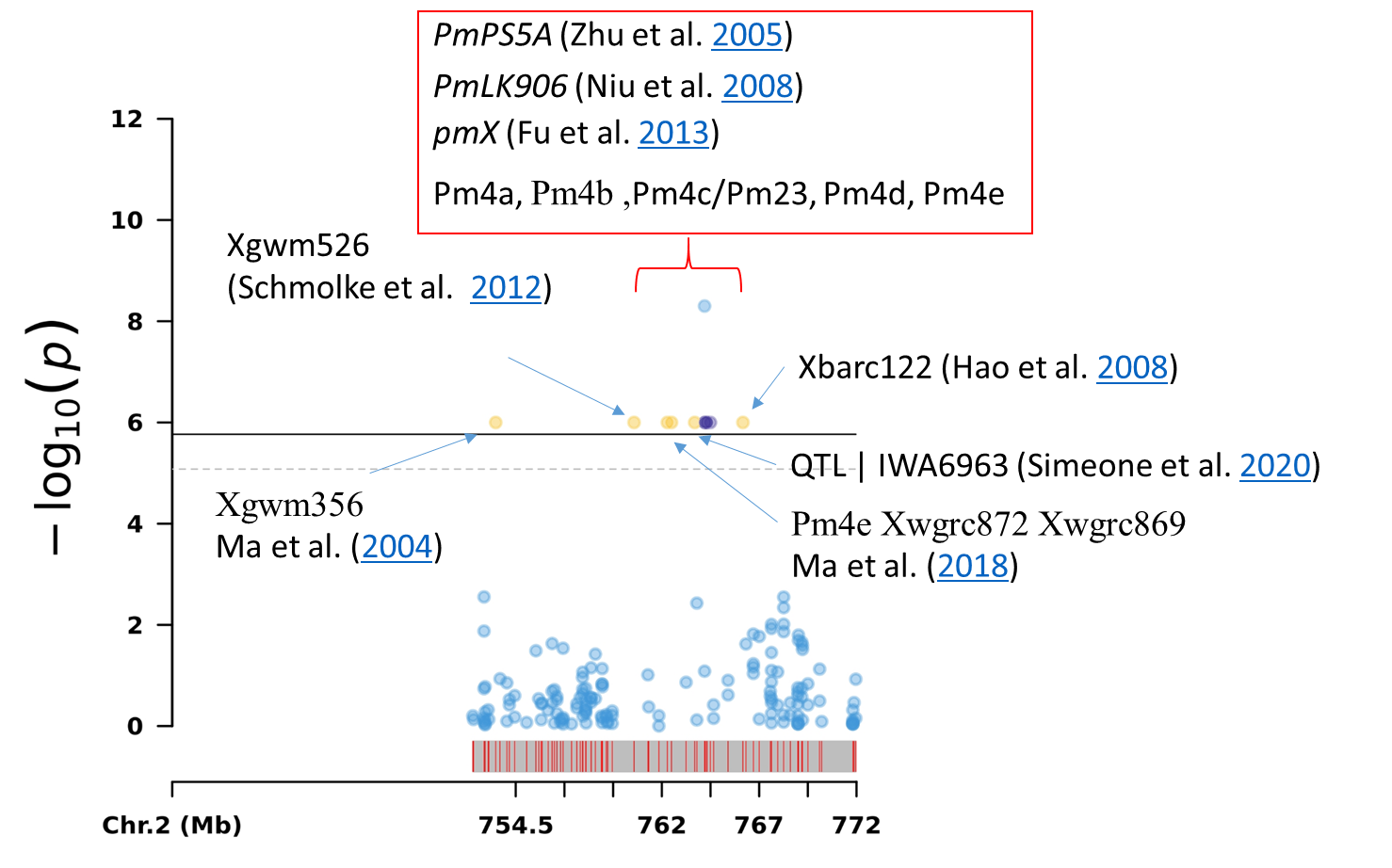


Figure S1: Published SSR-Marker reffering to published resistance gene in the MTA-5 Region


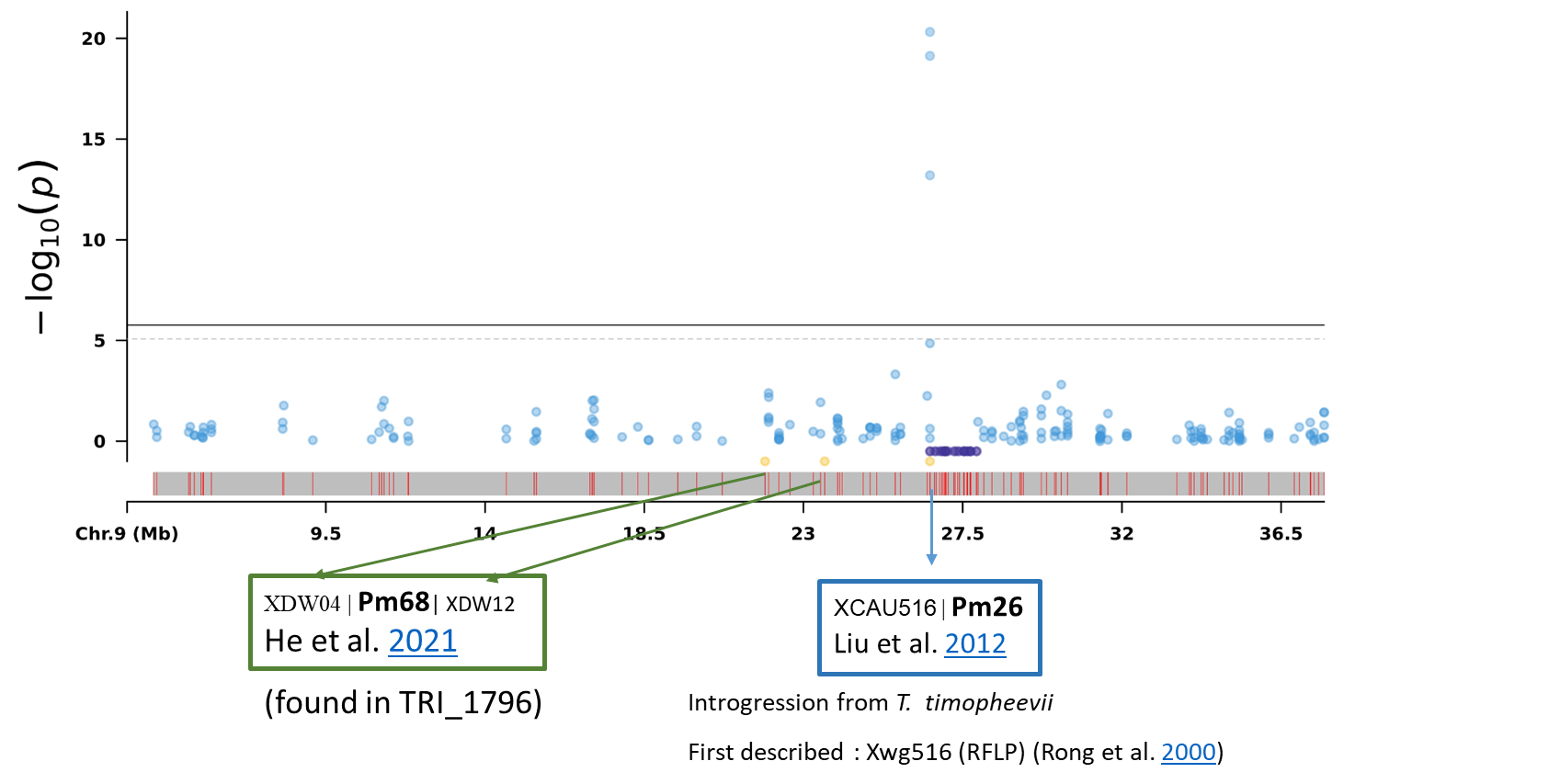


Figure S2: Published SSR-Marker reffering to published resistance gene (Pm26) in the MTA-30 Region
